# A case report of X-linked hypophosphatemia combined with primary hyperparathyroidism

**DOI:** 10.3389/fendo.2025.1634377

**Published:** 2025-07-29

**Authors:** YinQiong Wu, Min Yang, Ying Zhang, Qin Wang

**Affiliations:** Department of Endocrinology and Metabolism, West China Hospital of Sichuan University, Chengdu, Sichuan, China

**Keywords:** hypophosphatemic osteomalacia, X-linked hypophosphatemia, primary hyperparathyroidism, parathyroidectomy, bone pain

## Abstract

Both hypophosphatemic osteomalacia and primary hyperparathyroidism (PHPT) can lead to hypophosphatemia, but their simultaneous occurrence in the same patient is exceedingly rare. This article reports a case of a 43-year-old female patient whose primary clinical manifestations included pain in the lumbosacral and scapular regions, restricted mobility, and biochemical findings of decreased serum phosphate levels with normal parathyroid hormone (PTH) levels. The patient’s symptoms improved after treatment with active vitamin D supplementation, although neutral phosphate supplements were not administered. Eight years later, the patient’s symptoms progressively worsened. Further investigations revealed elevated PTH levels and worsening hypophosphatemia. Neck contrast-enhanced ultrasonography and parathyroid radionuclide imaging both indicated nodules in the right parathyroid gland. Postoperative pathological examination confirmed the diagnosis of parathyroid adenoma. Whole-exome sequencing identified a heterozygous deletion in exon 11 of the PHEX gene, consistent with a diagnosis of X-linked hypophosphatemia (XLH).

## Introduction

Hypophosphatemic Osteomalacia is a rare metabolic bone disorder characterized by impaired bone mineralization caused by hypophosphatemia. In adults, the primary clinical manifestations include fatigue, bone pain, skeletal deformities, reduced height, multiple fractures, and restricted mobility. Pathological fractures may occur even with minimal trauma. According to the literature, the incidence of hypophosphatemic osteomalacia is approximately 3.9 per 100,000 individuals (1).

Primary Hyperparathyroidism (PHPT) is an endocrine disorder characterized by excessive secretion of PTH. The typical biochemical features include hypercalcemia and elevated PTH levels. The prevalence of PHPT in the general population is estimated to range from 1 in 500 to 1 in 1,000 individuals. The main clinical manifestation of PHPT is progressively worsening generalized bone pain, and surgical removal of abnormal parathyroid tissue is the only definitive treatment for this condition (2).

Although there are well-established diagnostic and treatment guidelines for Hypophosphatemic Osteomalacia and PHPT as individual diseases, the simultaneous occurrence of these two conditions in a single patient is exceedingly rare. This not only increases the complexity of diagnosis but also poses challenges in selecting appropriate treatment strategies. Such uncommon coexistence of conditions is scarcely reported in the medical literature.

## Case presentation

The patient, a 43-year-old female, was admitted to our hospital, with the chief complaint of “lumbosacral pain accompanied by lower limb weakness for 10 years.” Skeletal deformities of the lower extremities and growth retardation that resulted in his present short stature (1.48 m) were immediately apparent. Approximately 10 years prior (33 years old), the patient developed unexplained bilateral lumbosacral, scapular, and knee joint pain, accompanied by lower limb weakness, with difficulty in activities such as squatting, turning over, and standing up.

**Figure 1:**
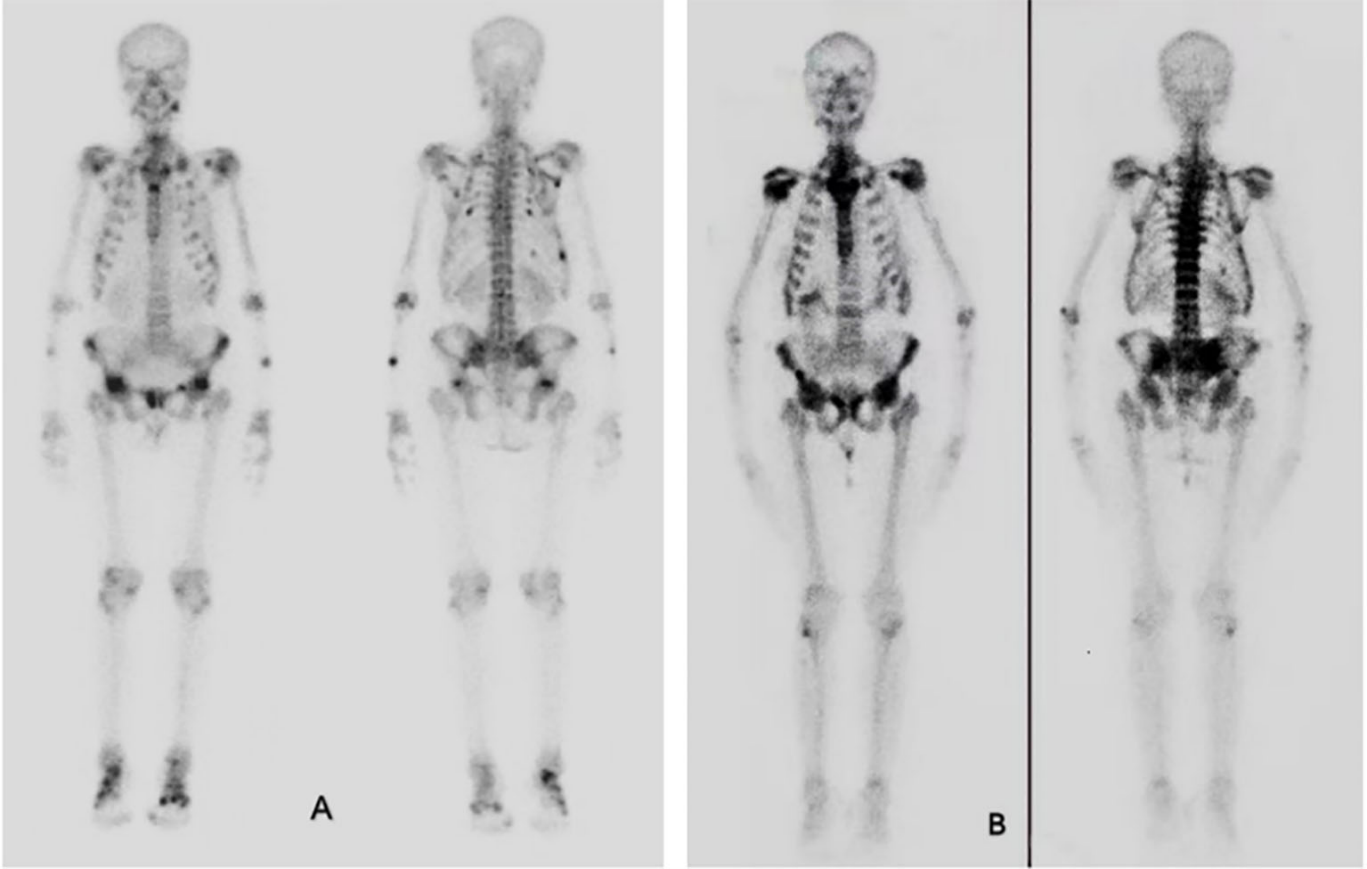
Bone scintigraphy. **(A)** Bone scintigraphy 10 years ago showed increased metabolism in whole body bones and joints, and increased spot-like metabolism in ribs, clavicle and pelvis, which was consistent with osteomalacia. **(B)** Bone scintigraphy in 2023 showed obvious concentration of axial bone nuclides in the whole body, with typical necktie sign and beaded rib changes. Nuclide concentration was evident in shoulder joints, pelvis, sacroiliac joints and spine.

Eight years ago, due to symptom progression, the patient (aged 35 years old) was hospitalized. The patient presents with low back, hip pain, and difficulty in squatting, turning over, and standing up. Chest compression test is positive. Laboratory investigations revealed significant hypophosphatemia, vitamin D deficiency, a high bone turnover rate, and markedly reduced bone mineral density (showed in **Tables 1**, **2**), while parathyroid hormone (PTH) levels remained normal. Whole-body SPECT bone imaging demonstrated multiple rib fractures throughout the body, as well as increased radiotracer uptake in the joint areas and calcaneal bones (see **Figure 1A**). Chest x-ray showed a fracture of the posterior ramus of the 4th-7th rib on the right side (see **Supplementary Figure S1**). Plain radiographs of the pelvis and thoracic spine showed slightly blurred trabecular bone patterns (see **Supplementary Figure S2**, **S3**).

**Table 1:** Laboratory results of two hospital admissions and follow-up visits.

Laboratory parameters	Reference range	10-yr ago	Before surgery	1d after surgery	3m after surgery	6m after surgery
Albumin-corrected serum calcium (mmol/L)	2.11-2.52	2.08-2.29	2.36-2.62	2.20	2.45	2.26
phosphorus(mmol/L)	0.85-1.51	0.55-0.69	0.29-0.37	0.51	0.52	0.47
bALP(ug/l)	11.4∼24.6	31.12	42.03	NA	NA	32.36
β-ctx (ng/ml)	0.03-0.568	NA	0.159	0.087	NA	0.092
P1NP (ng/ml)	8.53-64.32	NA	70.70	139	NA	42.60
PTH (pmol/L)	1.60-6.90	3.47	34.26	3.12	5.41	5.72
Serum 25-OHD (nmol/l)	75-125	44.39	18.1	NA	101	91.4
Serum creatinine (umol/L)	48-79	36	46	NA	NA	NA
eGFR ml/min/1.73m2	>90	147.21	128.12	NA	NA	NA
24hr Urine calcium (mmol)	2.5-7.5	1.08	3.41	NA	NA	NA
24hr Urine phosphorus (mmol)	22-48	6.12	10.34	NA	NA	NA

bALP, Bone alkaline phosphatase; β-CTX, Beta-collagen degradation products; P1NP, Total type I collagen amino terminal extension peptide; PTH, Parathyroid hormone; 25-OHD, 25 hydroxyvitamin D; eGFR, Estimate glomerular filtration rate.

Renal function was stable and creatinine was not repeated.

**Table 2:** Bone mineral density(g/cm^2^) and Z score follow-up results of the patient.

Region	10 years ago(Age 33)	Pre-surgery(Age 43)	6m-post-surgery(Age 43)	Change%
Lumber spine 1-4	0.883/-1.1	0.631/-3.3	0.864/-2.1	+36.9%
Femoral neck	0.730/-1.1	0.457/-3.2	0.611/-2.7	+33.7%
Total hip	0.707/-1.6	0.442/-3.5	0.599/-2.9	+35.5%

The above tests are performed on the same machine.

The patient was diagnosed with hypophosphatemic osteomalacia. She was discharged after receiving long-term treatment with oral calcium citrate(600 mg, once daily) and alfacalcidol (0.5 μg, once daily). One month after discharge, her symptoms began to alleviate, and the ability to perform daily activities returned to normal gradually. She was able to walk continuously for half a day without difficulty.

Two years ago (aged 41 years old), the patient began experiencing recurrent weakness in both lower limbs after prolonged walking. The condition progressively worsened, resulting in significant difficulty in performing activities such as turning over, sitting up, and squatting. She also presented with a noticeable limp during walking, accompanied by pain and discomfort in the bilateral lumbosacral region and thoracic cage. Notably, since the onset of her illness, the patient had never received neutral phosphate supplements. Nine months prior to admission, the patient discontinued calcium carbonate and alfacalcidol without medical supervision, leading to a rapid worsening of symptoms and subsequent hospital admission. The patient denied using any medications other than calcium carbonate and alfacalcidol and reported no family history of similar disorders. Additionally, the patient exhibited no symptoms of tendinopathy.

## Physical examination upon admission

The patient’s vital signs were stable, and she exhibited a limping gait. The Short Physical Performance Battery (SPPB) score was 7, suggesting impaired physical function. Physical examination revealed no thyroid enlargement, no spinal deformities, and no tenderness in the spine, with a normal range of motion. Muscle strength was normal in the upper limbs (Grade V) but reduced in the lower limbs (Grade IV), with normal muscle tone in all extremities. Significant tenderness was noted in the bilateral thoracic cage and lumbosacral regions. Overall, the findings were indicative of osteomalacia.

## Laboratory tests

The results of laboratory tests are presented in **Table 1**, showing hypercalcemia, hypophosphatemia, elevated parathyroid hormone levels, and significantly increased bone turnover. Bone mineral density (BMD) had significantly declined compared to the previous examination (see **Table 2**). These findings support a diagnosis of primary hyperparathyroidism (PHPT).

Additional laboratory evaluations, including routine blood, urine, and stool tests, hepato-renal function tests, serum immunological examination, thyroid function tests, and blood gas analysis, were all normal. Both serum protein electrophoresis and immunofixation electrophoresis yielded negative results, effectively ruling out other metabolic bone disorders and secondary osteoporosis. ​Fibroblast growth factor 23 (FGF23) testing was not performed due to limited laboratory resources. Add a table of differential diagnoses for chronic hypophosphatemia with corresponding markers to give more depth to the literature.

## Imaging findings

Thyroid contrast-enhanced ultrasound revealed a nodule measuring 13 × 4 × 8 mm on the deep surface of the middle portion of the right thyroid lobe. The characteristics observed on contrast-enhanced imaging strongly suggested a parathyroid adenoma.

99mTc-methoxy-isobutyl-isonitrile (99mTc-MIBI) parathyroid scintigraphy with fused CT scan demonstrated localized increased uptake in a nodule (11 × 6 × 9 mm) posterior to the middle portion of the right thyroid lobe, indicative of a hyperfunctioning parathyroid lesion.

Whole-body bone scintigraphy revealed multiple areas of increased metabolic activity, including the ribs, bilateral shoulder joints, bilateral knee joints, multiple thoracic vertebrae, and the pelvis. These findings showed significantly intensified tracer uptake compared to the scintigraphy results from eight years prior (**Figures 1A, B**).

## Treatment

The diagnosis of primary hyperparathyroidism (PHPT) was established based on the findings of hypercalcemia, hypophosphatemia, markedly elevated PTH levels, and imaging results from parathyroid fusion imaging and contrast-enhanced ultrasound. Then the patient underwent a ‘right inferior parathyroid adenoma resection,’ and postoperative pathological examination revealed parathyroid adenoma.

Postoperatively, the patient was treated with calcium citrate, vitamin D3, and calcitriol. Calcium supplementation was initiated to counteract the patient’s low-calcium diet and bone hungry syndrome after adenoma resection, which would have impeded bone mineralization recovery. Follow-up tests showed that PTH and serum calcium levels returned to normal, while serum phosphate increased but did not fully normalize. A further phosphorus clearance test revealed a TmP/GFR value of 0.86 (normal range: 0.85–0.95), which is on the lower end of the normal spectrum.

## Post-discharge follow-up

At discharge, the patient reported some relief from lumbosacral and thoracic pain (Visual Analogue Scale score: 5), and both knee pain and fatigue had significantly reduced. The SPPB score improved from 7 on admission to 8, reflecting a notable improvement in the patient’s overall physical function.

During the follow-up period after discharge, the patient continued receiving calcium citrate, vitamin D3, and calcitriol. Burosumab (a monoclonal antibody against FGF23) was not administered because the medication was unavailable in the patient’s region.

At 3 months postoperatively, the patient’s serum calcium, PTH, and 25-hydroxyvitamin D (25-OHD) levels had returned to normal, but serum phosphate levels had not fully normalized (see **Table 1**). The patient reported some relief from lumbosacral pain (VAS score: 3), but knee pain persisted.

At 6 months postoperatively, serum calcium and PTH levels remained normal. Bone turnover markers showed a decrease compared to preoperative levels, and bone mineral density measurements demonstrated significant improvement, reflecting enhanced bone health. However, serum phosphate levels remained subnormal (see **Table 1**). The patient reported significant relief from knee pain (VAS score: 1) and was able to complete normal physical activity without pain. The SPPB score reached the maximum of 12.

## Genetic testing

Due to the patient’s long-standing hypophosphatemia, peripheral blood was collected for whole exome sequencing with the patient’s consent. A heterozygous deletion of exon 11 in the PHEX gene was detected, and diagnosis of X-linked dominant hypophosphatemic osteomalacia was confirmed in December 2023. As she had no children, genetic testing in offspring was not performed.

## Discussion

The patient initially presented with insidious onset of generalized bone pain and bilateral lower limb weakness, limiting mobility. Laboratory tests showed persistent hypophosphatemia, and whole exome sequencing revealed a heterozygous deletion at exon 11 of the PHEX gene, confirming X-linked hypophosphatemia (XLH). After treatment with calcium carbonate and alfacalcidol, the symptoms fully resolved, and daily activity returned to normal. Eight years later, the patient experienced a recurrence of lower limb weakness, bone pain, and worsened mobility. Laboratory tests showed elevated PTH and calcium levels, decreased serum phosphate, and reduced bone mineral density. Parathyroid scintigraphy and ultrasound identified a nodule in the right thyroid lobe. Postoperative pathology confirmed a parathyroid adenoma, leading to the diagnosis of XLH with primary hyperparathyroidism.

The co-occurrence of hypophosphatemic osteomalacia with secondary or tertiary hyperparathyroidism is a well-documented phenomenon (3, 4). In contrast, the coexistence of primary hyperparathyroidism and hypophosp hatemic osteomalacia is uncommon, particularly in patients with hypophosphatemic osteomalacia who have not received phosphate supplementation. A thorough literature review revealed only two similar cases. The first case was reported in 1995 by Jergen Knudtzon: a 46-year-old female, was diagnosed with hypophosphatemic osteomalacia at the age of 2 and began receiving high-dose ergocalciferol treatment (5). The second was reported by S.T. Tournis et al. in 2005 describing a 56-year-old male XLH patient with lower limb skeletal deformities and short stature from childhood, but who had not received neutral phosphate supplementation treatment for XLH (6). The comparison of three cases were listed in **Table 3**. All three patients without a history of phosphate supplements.

**Table 3:** comparison of three cases of XLH combined with HPT.

Comparative Dimension	Knudtzon (5)	Tournis (6)	Current Case
Diagnostic Background	XLH diagnosed in childhood	XLH diagnosed in childhood	XLH confirmed by gene testing in adulthood
Treatment History	Long-term vitamin D_2_ treatment	No phosphate or vitamin D treatment	Active vitamin D treatment (no phosphate)
Age at PHPT Diagnosis	29 years (after 27 years of XLH)	59 years	43 years (after 10 years of XLH)
PHPT Diagnosis	Elevated PTH, hypercalcemia, imaging abnormalities	Elevated PTH, hypercalcemia, imaging abnormalities	Elevated PTH, hypercalcemia, imaging abnormalities
Type of Parathyroid Lesion	Hyperplasia	Hyperplasia	Adenoma (pathologically confirmed)
Postoperative Outcome	Normal calcium, elevated PTH	PTH near normal, partial phosphate recovery	Normal PTH, incomplete phosphate recovery

Due to the mutation of the PHEX gene, XLH leads to an abnormal increase in fibroblast growth factor 23 (FGF23). FGF23 causes a decrease in blood phosphorus by inhibiting phosphate reabsorption in the proximal tubule and phosphate absorption in the intestine. At the same time, it inhibits 1α-hydroxylase and reduces the production of active vitamin D (7). Supplementation of phosphate and active vitamin D constitutes the conventional treatment for XLH. The reason our patient did not receive phosphate supplementation is that our hospital lacks neutral phosphate. Calcium supplementation therapy was initiated because the patient has been on a long-term low-calcium diet. Research has found that Prolonged supplementation of phosphate may result in secondary hyperparathyroidism even tertiary hyperparathyroidism. Prolonged excessive phosphate supplementation can cause phosphate to bind with calcium, forming insoluble calcium phosphate complexes, which subsequently reduce calcium absorption in the gastrointestinal tract. Moreover, intermittent spikes in serum phosphate levels induced by such over-supplementation can trigger the release of PTH, potentially resulting in the progression to tertiary hyperparathyroidism (8, 9). High levels of PTH not only further reduce blood phosphorus, but also directly activate the PTH1R receptor of bone cells and promote FGF23 synthesis (10). In addition, to prevent hypocalcemia in patients and promote the increase of bone density, we used calcium supplements, but this might aggravate hypophosphatemia. On the other hand, hypophosphatemia caused by excessive FGF23 may stimulate PTH (11).

However the association between XLH and primary hyperparathyroidism remains unclear, studies by Blydt-Hansen TD et al. have shown that PHEX is widely expressed in the parathyroid glands, and PHEX dysfunction may impair the degradation of PTH, resulting in increased PTH level (12). Other studies. suggest that loss of PHEX function could lead to abnormal cleavage or degradation of PTH mRNA, contributing to the development of hyperparathyroidism.

At present, inhibition of FGF23 activity is considered as a new method for the treatment of XLH. Burosumab is an IgG1 monoclonal antibody that specifically recognizes the N-terminal region of FGF23 and neutralizes FGF23, thereby restoring tubular phosphorus reabsorption and increasing active vitamin D levels. Burosumab has been shown to improve biochemical abnormalities, radiographic signs of rickets, impaired growth, fracture healing, and mineralization in patients with XLH (13).

Thus, for patients with hypophosphatemic osteomalacia, especially those receiving neutral phosphate supplementation, regular monitoring of serum calcium, phosphate, and parathyroid hormone levels is crucial. When serum calcium and PTH levels increase, clinicians should be alert not only to the possibility of secondary or tertiary hyperparathyroidism but also to the rare occurrence of primary hyperparathyroidism.

## Data Availability

The datasets presented in this study can be found in online repositories. The names of the repository/repositories and accession number(s) can be found in the article/**Supplementary Material**.

## References

[1] Xu C Zhao J Xia W. Guidelines for diagnosis and treatment of hypophosphatemic rickets/osteomalacia in China. Chin J Osteoporosis Bone Mineral Salt Dis. (2022) 15:107–25. doi: 10.3969/j.issn.1674-2591.2022.02.001

[2] Guidelines for diagnosis and treatment of primary hyperparathyroidism. Chin J Osteoporosis Bone Mineral Salt Dis. (2014) 7:187–98. doi: 10.3969/j.issn.1674-2591.2014.03.002

[3] Tournis S Georgoulas T Zafeiris C Papalexis C Petraki K Lyritis GP. Tertiary hyperparathyroidism in a patient with X-linked hypophosphatemic rickets. J Musculoskelet Neuronal Interact. (2011) 11:266–9. doi: 10.1530/boneabs.7.p82, PMID: 21885902

[4] DeLacey S Liu Z Broyles A El-Azab SA Guandique CF James BC. Hyperparathyroidism and parathyroidectomy in X-linked hypophosphatemia patients. Bone. (2019) 127:386–92. doi: 10.1016/j.bone.2019.06.025, PMID: 31276850 PMC6836672

[5] Knudtzon J Halse J Monn E Nesland A Nordal KP Paus P. Autonomous hyperparathyroidism in X-linked hypophosphataemia. Clin Endocrinol (Oxf). (1995) 42:199–203. doi: 10.1111/j.1365-2265.1995.tb01863.x, PMID: 7704964

[6] Tournis ST Giannikou PV Paspati IN Katsalira EA Voskaki IC Lyritis GP. Co-existence of X-linked hypophosphatemic rickets (XLH) and primary hyperparathyroidism: case report and review of the literature. J Musculoskelet Neuronal Interact. (2005) 5:150–4. doi: 10.1097/ten.0b013e3181514e2b, PMID: 15951631

[7] Takashi Y Kawanami D Fukumoto S. FGF23 and hypophosphatemic rickets/osteomalacia. Curr Osteoporos Rep. (2021) 19:669–75. doi: 10.1007/s11914-021-00709-4, PMID: 34755323

[8] Firth RG Grant CS Riggs BL. Development of hypercalcemic hyperparathyroidism after long-term phosphate supplementation in hypophosphatemic osteomalacia. Rep Two Cases Am J Med. (1985) 78:669–73. doi: 10.1016/0002-9343(85)90411-5, PMID: 2984933

[9] Jeon HJ Kwon SH Kim SW Shin CS Park KS Kim SY. Evaluation of the parathyroid function in six patients with hypophosphatemic osteomalacia, including a case of tertiary hyperparathyroidism developing during combined oral phosphate and vitamin D therapy. Horm Res. (2003) 60:127–33. doi: 10.1159/000072524, PMID: 12931040

[10] Fan Y Bi R Densmore MJ Sato T Kobayashi T Yuan Q. Parathyroid hormone 1 receptor is essential to induce FGF23 production and maintain systemic mineral ion homeostasis. FASEB J. (2016) 30:428–40. doi: 10.1096/fj.15-278184, PMID: 26428657 PMC4684518

[11] Carpenter TO Insogna KL Zhang JH Ellis B Nieman S Simpson C. Circulating levels of soluble klotho and FGF23 in X-linked hypophosphatemia: circadian variance, effects of treatment, and relationship to parathyroid status. J Clin Endocrinol Metab. (2010) 95:E352–7. doi: 10.1210/jc.2010-0589, PMID: 20685863 PMC2968736

[12] Blydt-Hansen TD Tenenhouse HS Goodyer P. PHEX expression in parathyroid gland and parathyroid hormone dysregulation in X-linked hypophosphatemia. Pediatr Nephrol. (1999) 13:607–11. doi: 10.1007/s004670050669, PMID: 10460513

[13] Haffner D Emma F Seefried L Högler W Javaid KM Bockenhauer D. Clinical practice recommendations for the diagnosis and management of X-linked hypophosphataemia. Nat Rev Nephrol. (2025) 21:330–54. doi: 10.1038/s41581-024-00926-x, PMID: 39814982

