# Supplementary material for: A case report of X-linked hypophosphatemia combined with primary hyperparathyroidism
Source: Front Endocrinol (Lausanne). 2025 Jul 29;16:1634377. doi: 10.3389/fendo.2025.1634377 (PMC12339324; doi:10.3389/fendo.2025.1634377)
Supplement: Supplementary file 1 [file Table1.docx]

**Supplementary Table1.** Differential diagnoses for chronic hypophosphatemia with corresponding markers

| **Classification of etiology** | **Common diseases** | **Key markers/checks** |
| --- | --- | --- |
| Inadequate intake | Long-term malnutrition  alcoholism  parenteral nutrition without phosphorus supplementation | Serum phosphorus↓, urine phosphorus↓, albumin↓, body weight↓ |
| Intestinal absorption reduced | Vitamin D deficiency, malabsorption syndrome (celiac disease) | Serum 25-OH vitamin D↓, 1,25-(OH)₂ vitamin D↓, fecal lipid ↑，多种血电解质和白蛋白降低 |
| Increased kidney losses | x-linked hypophosphatemia, Tumor-induced osteomalacia | FGF23↑, Urine phosphorus ↑,  TmP /GFR↓ |
|  | Fanconi syndrome | Urine phosphorus ↑, urine glucose ↑, urine amino acids ↑, blood uric acid ↓ |
|  | Secondary hyperparathyroidism (chronic kidney disease) | PTH ↑, serum calcium ↓ or normal, eGFR ↓ |
| Endocrine abnormalities | Primary hyperparathyroidism | PTH ↑, blood calcium ↑, urine phosphorus ↑ |
|  | Diabetic ketoacidosis | Blood glucose ↑, blood ketones ↑, serum phosphorus ↓after insulin treatment |
| Phosphate shift into cells​ | Refeeding syndrome | Blood phosphorus ↓ (fast), blood potassium ↓, blood magnesium ↓ |
| Drugs or toxins | Long-term diuretics, excessive antacids | Urine phosphorus ↑ (diuretic), blood aluminum ↑ (aluminum-containing antacid) |
