# Supplementary figures and images for: A case report of X-linked hypophosphatemia combined with primary hyperparathyroidism
Source: Front Endocrinol (Lausanne). 2025 Jul 29;16:1634377. doi: 10.3389/fendo.2025.1634377 (PMC12339324; doi:10.3389/fendo.2025.1634377)

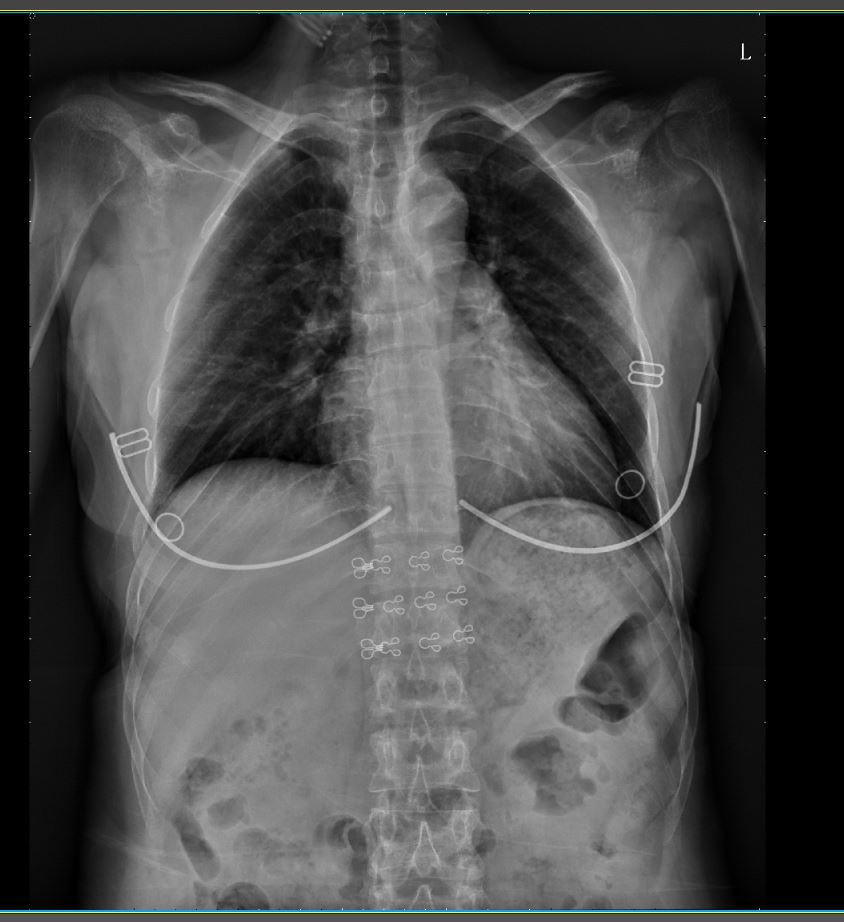

Supplement: Supplementary file 3 [file Image1.jpeg]

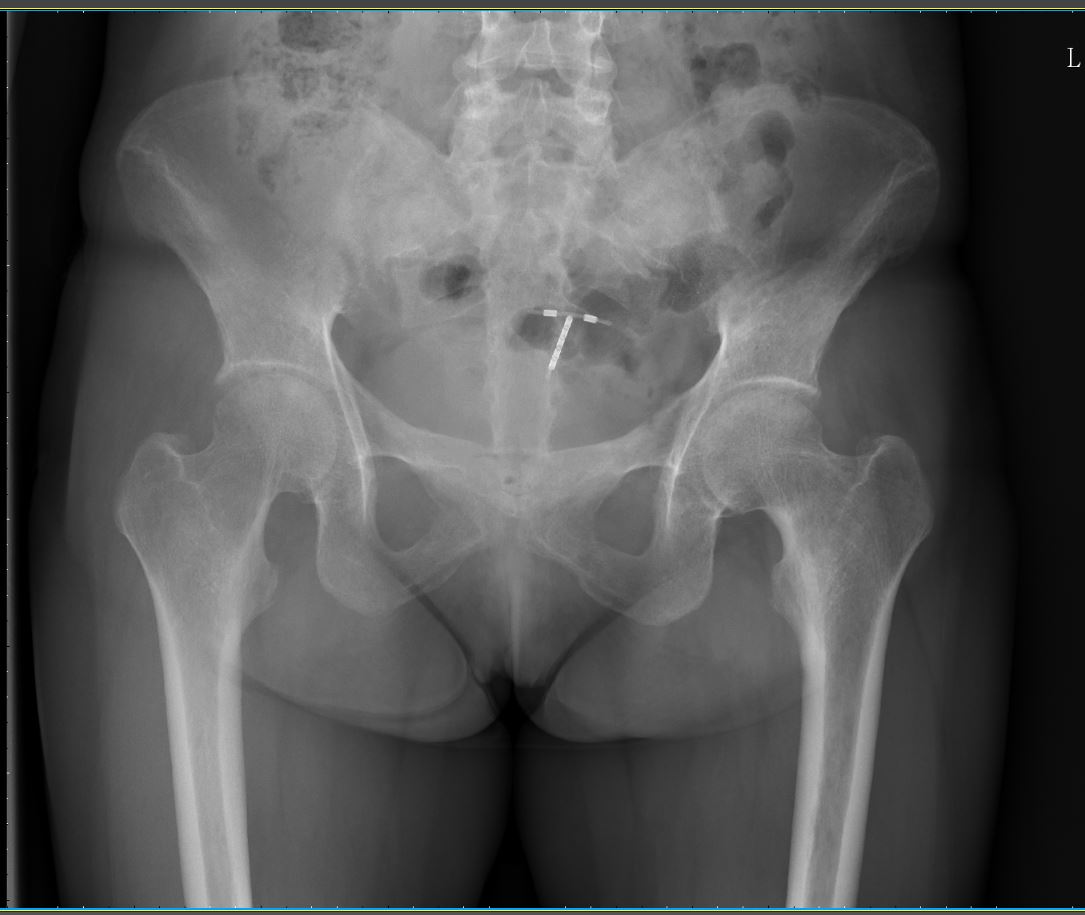

Supplement: Supplementary file 4 [file Image2.jpeg]

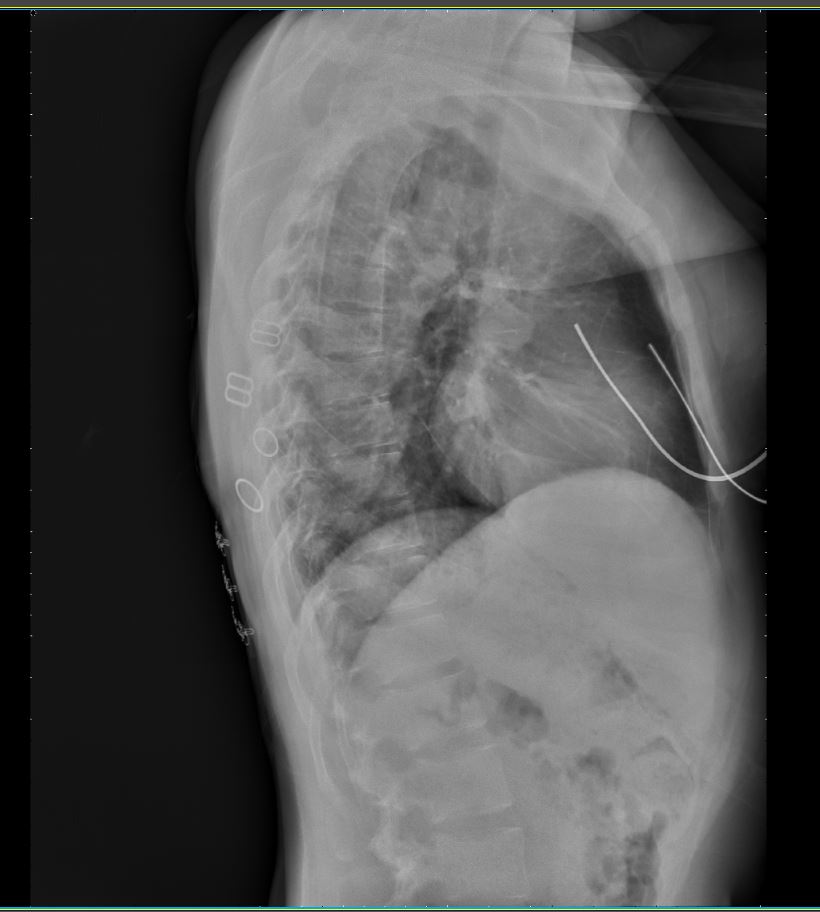

Supplement: Supplementary file 5 [file Image3.jpeg]
